# Large enchondroma of the thoracic spine: a rare case report and review of the literature

**DOI:** 10.1186/s12891-017-1519-z

**Published:** 2017-04-13

**Authors:** Jing Guo, Ju-zhou Gao, Lian-jin Guo, Zhi-xun Yin, Er-xing He

**Affiliations:** 1 Spine Surgery, The First Affiliated Hospital of Guangzhou Medical University, 151 Yanjiang West Road, Guangzhou, 510000 China; 2 Guangzhou Orthopaedic Institute, Guangzhou, China

**Keywords:** Enchondroma, Thoracic spine, Chondroma, Case report

## Abstract

**Background:**

Enchondroma, a subtype of chondroma, originates from the medullary cavity of the bone and produces an expansile growth pattern. Enchondroma located in the spine is rare and a few cases of large thoracic enchondroma have been reported. The authors document a rare case of large enchondroma in the thoracic spine of a 49-year-old woman, and discuss its clinical, radiological and histopathological characteristics.

**Case presentation:**

The patient presented with rapidly progressive and severe pain on her upper back. Magnetic resonance imaging revealed an expansile lesion at the posterior elements of T3 that was hypointense on T1-weighted images and mixed iso- to hyperintense on T2-weighted images. Administration of gadolinium-diethylenetriamine pentaacetic acid (Gd-DTPA) resulted in heterogeneous enhancement. During surgery, a large tumor of 4.2cm × 4.7cm × 2.1cm was resected along with the lamina and spinous process. Histological examination revealed that the tumor consisted of mature hyaline cartilage with typical chondrocytes, indicating that it was an enchondroma.

**Conclusions:**

Despite its benign-growing nature, enchondroma should be examined closely for signs of enchondromatosis and enchondrosarcoma. Complete surgical resection is the treatment of choice for immediate relief of symptoms and avoidance of recurrence.

## Background

Chondroma is a slowly growing benign cartilaginous tumor, and rarely affects the spine [1]. According to its site of origin, chondroma can be subdivided into 2 groups: periosteal chondroma and enchondroma. Periosteal chondroma arises from the surface of periosteum and grows in an exophytic fashion [2]; whereas, enchondroma originates from the medullary cavity and produces an expansile growth pattern [3, 4]. Spinal chondroma accounts for only about 3% of all chondromas and has been noted to be commonly encountered in the thoracic region [1]. However, to the best of our knowledge, few report on large enchondroma in the thoracic spine exists in literatures. Here, we report on a 49-year-old woman with severe upper back pain due to a large enchondroma at the posterior elements of T3 vertebra.

## Case presentation

**Fig. 1:**
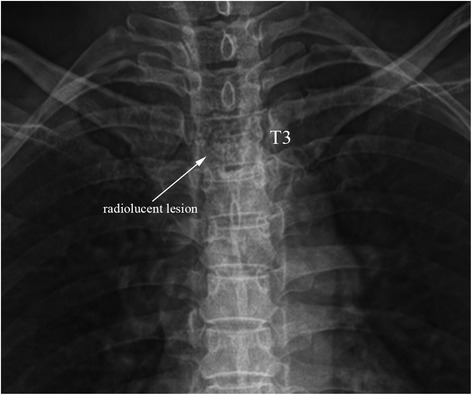
Preoperative anteroposterior view of the thoracic spine showing a radiolucent lesion at the spinous process of T3 vertebra (*white arrow*)

**Fig. 2:**
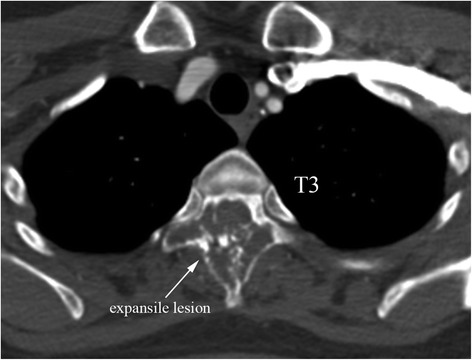
Preoperative axial CT scans showing an expansile lesion of osteolysis at the posterior elements of T3 vertebra, involving spinous process, lamina, right-side transverse process and pedicle (*white arrow*)

**Fig. 3:**
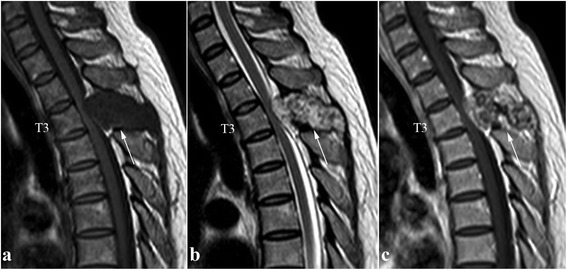
Preoperative sagittal T1-weighted (**a**) and T2-weighted (**b**) MR images showing a well-circumscribed extradural mass, which was homogeneous hypointense on T1-weighted images and mixed iso- to hyperintense on T2-weighted images. Administration of Gd-DTPA resulted in heterogeneous enhancement on T1-weighted images (**c**) (*white arrows*)

A 49-year-old woman presented with progressive and severe upper back pain for 1 month. Severe tenderness was detected over the T3 spinous process. There was no motor deficit, and only mild sensory loss distal to the right ankle. Plain radiographs of the thoracic spine showed a radiolucent lesion at the T3 spinous process (Fig. 1). Computed tomography (CT) revealed an expansile lesion of osteolysis at the posterior elements of T3, involving spinous process, lamina, right-side transverse process and pedicle (Fig. 2). Magnetic resonance imaging (MRI) showed a well-circumscribed mass, which was homogeneous hypointense on T1-weighted images (T1-WI) and mixed iso- to hyperintense on T2-weighted images (T2-WI) (Fig. 3a and b). Heterogeneous enhancement was observed after gadolinium-diethylenetriamine pentaacetic acid (Gd-DTPA) administration (Fig. 3c).

**Fig. 4:**
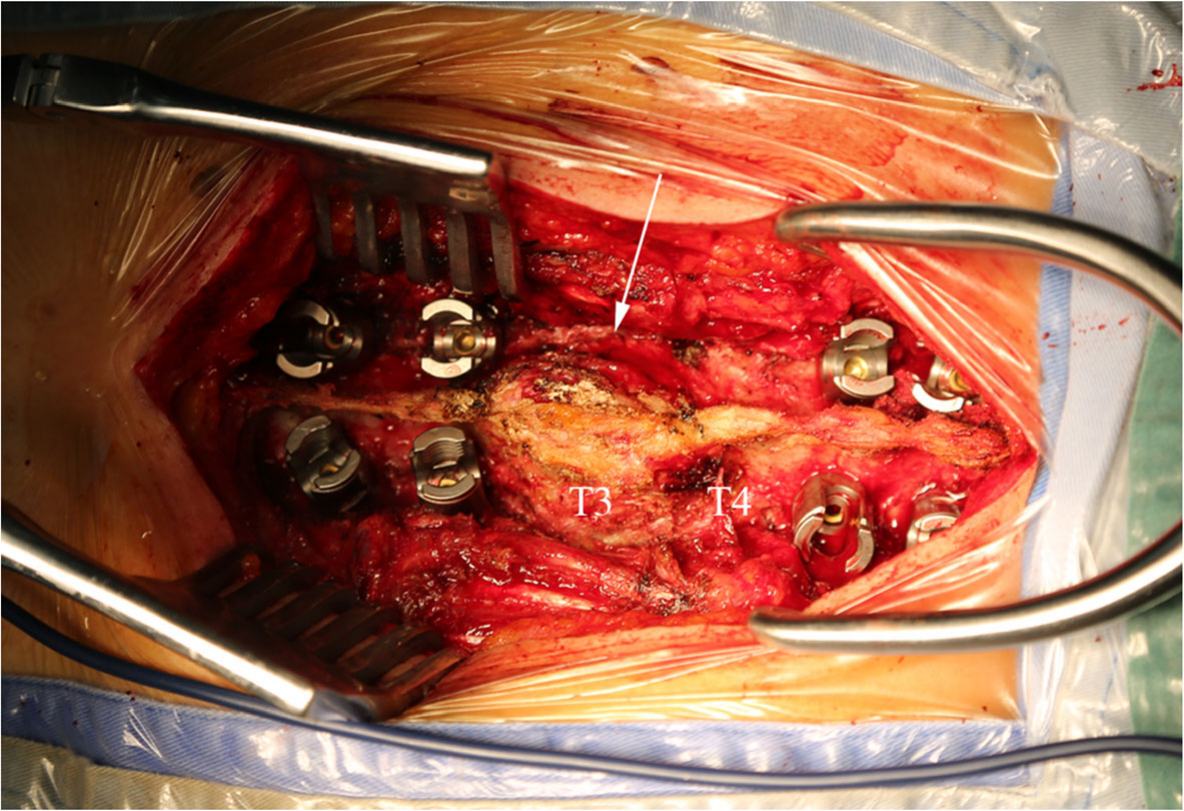
Intraoperative photograph showing an enormous mass involving the T3 spinous process as well as the superior part of T4 spinous process (*white arrow*)

**Fig. 5:**
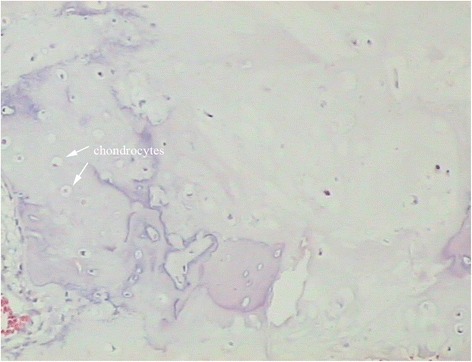
Histological examination showing mature hyaline cartilage with nests of benign-appearing chondrocytes (hematoxylin-eosin stain, original magnification × 200) (*white arrows*)

Excision of the tumor in situ was performed via a posterior approach. The mass was easily separated from the surface of the dura and measured in size of 4.2cm × 4.7cm × 2.1cm. The right transverse process was then removed, followed by curettage of the pedicle to maximally eliminate the residual tissues of the tumor. During surgery, we found that the superior part of T4 lamina and spinous process was compromised (Fig. 4). Therefore, laminectomy and removal of the spinous process at T4 was performed as well. Pedicle screw insertion and fixation was done at T1-T2 and T5-T6 segments to secure the stability of the spine. Grossly, the tumor was similar in appearance to lobules of mature cartilage, with several regions of grittiness scattered inside. Histological examination revealed that the tumor was comprised of abundant hyaline cartilage containing many nests of benign-appearing chondrocytes (Fig. 5). Small amount of calcium deposits were noted in the lacunae. No significant atypia or mitosis was seen. The pathological diagnosis was enchondroma without sarcomatous changes. Postoperative recovery was uneventful. Her upper back pain disappeared completely. The patient was ambulatory and tolerable to normal activity at the time of discharge two weeks after surgery. No adjuvant treatment was planned. At the 1-year follow-up, the patient showed no sign of recurrence.

## Discussion

Enchondroma is a slow-growing tumor and can develop at any part of the vertebra, including spinous process, lamina, pedicle, and vertebral body [1]. Nevertheless, posterior elements are more likely to be affected than other locations [5]. Enchondroma originates from the medullary cavity and usually grows inside the bone without cortex penetration [3, 4]. Neurological symptoms may develop gradually as the tumor grows and compresses the neural elements.

Enchondroma usually occurs in a solitary fashion [1]. The presence of multiple enchondromas is highly indicative of the diagnosis of enchondromatosis syndrome, such as Ollier disease and Maffucci syndrome [6, 7]. Ollier disease is a rare condition in which multiple enchondromas appear in the large and small tubular bones of limbs, usually with a unilateral predominance [6]. When associated with soft tissue hemangiomas, the disease is known as Maffucci syndrome [7]. Although the individual lesions are similar to solitary enchondromas, the risk of sarcomatous degeneration may be as high as 25% in patients with Ollier disease and Maffucci syndrome [1].

Plain radiographs of the spine are difficult to identify an enchondroma. CT scan is of help to clearly visualize the pathology and its relation to adjacent bones [3]. A radiolucent, erosive lesion can be revealed on a CT scan of bone setting. Stippled or scattered patterns of calcification inside the tumor may be present. MRI is useful in making diagnosis and distinguishing between benign and malignant lesions. Chandramohan et al [8] found that most chondromas demonstrated intermediate signal intensity on T1-weighted images and high signal intensity on T2-weighted images. Calcified cartilage with sporadic osseous content typically shows mixed low to high signal intensity on T2-weighted images. Peripheral rim enhancement on MR images after Gd-DTPA administration has been reported in chondromas as a feature for benign cartilaginous tumors [5]; however, in large chondromas, this phenomenon may not be apparent [2].

Grossly, enchondroma appears as lobules of firm, mature cartilage, with regions of grittiness signifying mineralization of the matrix [9]. Histologically, enchondroma is composed of neoplastic chondrocytes dispersed within an abundant hyaline or myxoid background [5]. The tumor cells may be arranged in a pseudolobular fashion, with foci of calcifications depositing in the lacunae. Exhibition of nuclear atypia and cellular mitosis is rare.

Surgical excision is generally recommended as the treatment of choice for cases with local and/or neurological symptoms. [1] The goal of surgery is to establish a histological diagnosis, prevent sarcomatous degeneration, and preserve neurological function. Recurrence of chondroma is less than 10% after surgery, and usually related with incomplete removal [2, 5, 10]. In cases in which excision results in spinal instability, the spine must be instrumented or reconstructed.

## Conclusion

We report a rare case of large enchondroma in the thoracic spine presenting severe upper back pain in a 49-year-old woman. Despite its benign-growing nature, enchondroma should be examined closely for signs of multiple enchondromatosis and enchondrosarcoma. Complete surgical resection is the treatment of choice for immediate relief of symptoms and avoidance of recurrence.

## Abbreviations

AP: Anteroposterior
CT: Computed tomography
Gd-DTPA: Gadolinium-diethylenetriamine pentaacetic acid
MRI: Magnetic resonance imaging
